# Sexual and reproductive health experience, knowledge and problems among university students in Ambo, central Ethiopia

**DOI:** 10.1186/s12978-017-0302-9

**Published:** 2017-03-14

**Authors:** Abenezer Yared, Zekariyas Sahile, Mulugeta Mekuria

**Affiliations:** 1 College of Social Sciences and Humanities, Ambo University, Ambo, Ethiopia; 2 Department of Public Health Officer, College of Medicine and Health Sciences, Ambo University, Ambo, Ethiopia

**Keywords:** SRH, STIs, Risk perception, Knowledge, Contraceptive methods, University students, Ethiopia

## Abstract

**Background:**

Youths in universities are at high risk of STIs and SRH problems in Ethiopia. However, students did not perceive themselves at risk of STI/HIV infection though reports showed they were sexually active, had multiple sexual partners and reported symptoms of STIs. Having recognized the threat posed by SRH problems, this study aimed to assess the SRH experiences, knowledge, and problems among university students at Ambo University in Ethiopia.

**Methods:**

A cross-sectional study was conducted in Ambo University main campus from January to February 2015 using mixed approach of quantitative (survey) and qualitative (in-depth interview) methods. Proportionate stratified sampling technique was used to select 400 survey respondents and purposive sampling was employed to identify 10 in-depth interviewees. The quantitative data was coded, entered to SPSS and descriptively analyzed, while the qualitative data was categorically organized, repeatedly reviewed and thematically analyzed.

**Results:**

Mean age during first sex of 17.29 ± SD 2.21 and mean number of past 12 months regular sexual partners of 1.36 ± SD 0.505 were recorded. Only 21.1% of survey respondents perceived themselves to be at risk of HIV. Almost all survey respondents ever heard of STIs (94.5%) and HIV/AIDS (98%), and 89.4% knew modern contraceptives such as pills (64.8%) and condoms (56.8%). Despite awareness of STIs including HIV/AIDS, more than one fifth (22.8%) had any of the STIs in the past one year. Although the quantitative data showed unwanted pregnancy (5%) and abortion (2.5%) existed in the campus minimally, high rates of unwanted pregnancy and unsafe abortion were reported in the qualitative data.

**Conclusions:**

SRH/STIs were problems among students of the university. Although students knew about STIs, the STI infection rate in the past year was quite high, and was almost as high as the percentage of students who reported sexual activity in the past year. Though reported by a minority of students, unwanted pregnancy and unsafe abortion may also be a problem. The university thus needs to launch a program directed towards STIs and SRH problems, particularly among female students.

## Plain english summary

There is an increasing concern about Sexual and Reproductive Health (SRH) problems in developing countries. In order to address the issue, this study explored university students’ SRH experiences, knowledge, and problems via survey and interview in Ambo, central Ethiopia. Of the 400 respondents, more than half (212) had an experience of sexual intercourse. Almost all surveyed students were aware of Sexually Transmitted Infections (STIs) including HIV/AIDS. Most of them also knew contraceptive methods such as pills and condoms. In spite of such awareness, 91 respondents told that they had some sort of STIs and interviewees said female students experienced unwanted pregnancy and abortion. In conclusion, although students knew about SRH, they failed to apply their knowledge to themselves and their sexual health. The university thus needs to launch an intervention program directed towards prevention of SRH problems, particularly among female students.

## Background

There is an increasing concern about Sexual and Reproductive Health (SRH) problems nowadays due to the adverse effect on the productive population of developing countries. In Ethiopia, millions of young people in general and adolescents and youths in Higher Education Institutions (HEIs) in particular are at high risk of Sexually Transmitted Infections (STIs) and SRH problems [1].

Previous studies carried out on the knowledge, attitudes and practices among youths with respect to HIV and other STIs indicated that significant number of students had sexual experience and/or were sexually active, had multiple sexual partners, reported symptoms of STIs, and experienced unwanted pregnancy and abortion [1–4]. Regarding risk perception, a study carried out in Addis Ababa University showed 80.7% of students did not perceive that they are at risk of STI/HIV [1].

Studies conducted among HEIs have reported on the factors contributing to SRH and STIs problems. These include substance abuse and addictions, surrounding hotspots, transactional sex, gender-based violence, sexual abuse and harassment, age, lack of life skills, peer pressure, early initiation of sex, adventurous and reckless behaviors and practices, exposure to new environment, absence of parental control and guidance, exposure to pornography and inadequate knowledge/information [3, 5, 6]. The fact that students’ SRH needs received little attention in HEIs also greatly contributed to the increased risk of these young people to the problems. Even the available SRH care was not known by and youth friendly to many students [1].

Even if some studies aimed at addressing the existing issue [2, 4, 6–9], most of the previous studies that investigated on this problem used only the quantitative methods as opposed to the present study which combined both qualitative and quantitative approaches. In an attempt to fill this gap and having recognized the threat posed by SRH problems on university youths that are expected to play a key role in their country’s development, the present study therefore aimed to assess SRH experiences, knowledge and problems among Ambo University (AU) students in central Ethiopia.

## Methods

### Design and setting

A cross-sectional study was conducted in AU main campus from January to February 2015 by utilizing mixed approach consisting of quantitative (survey) and qualitative (in-depth interview) methods. Established in 1939 as one of the oldest HEIs in Ethiopia, AU is a public university found in Ambo, a town located 114 km west of the capital Addis Ababa. The University runs 9 colleges/institutes/schools and 37 academic departments. Besides its main campus at Ambo, AU also has three other campuses – Awaro, Guder and Woliso. A total of 7,093 (4,965 male and 2,128 female) students coming from all over Ethiopia are enrolled in the university. Illness that would make students unable to respond questions was the exclusion criterion used in the study.

### Sampling

In calculating the sample size for the quantitative survey, it was assumed that the reasonable estimate of the proportion required to assess SRH experiences, knowledge and problems of the students (p) = q = 0.5. Other assumptions made were 5% marginal error (d) and confidence interval of 95% ($$ z\raisebox{1ex}{$\upalpha $}\!\left/ \!\raisebox{-1ex}{$2$}\right.=1.96 $$). The sample size (n) was given by $$ \frac{{\mathrm{z}}^2\mathrm{pq}}{{\mathrm{d}}^2} $$ which yielded 384.16. However, since the source population (all students of AU in 2014/15 academic year, N) was 7,093, the sample size correction formula to determine the final sample size (nf) for population <10,000 $$ \left(\mathrm{nf}=\frac{\mathrm{n}}{1+\left(\raisebox{1ex}{$\mathrm{n}$}\!\left/ \!\raisebox{-1ex}{$\mathrm{N}$}\right.\right)}\right) $$ was used to give 364. Hence, considering 10% non-response rate, sample size of the study was 400. Based on the university registrar’s enrollment roster, proportionate stratified sampling technique was utilized to determine number of students selected from each department in each year of study. Survey respondents from each stratum were then identified by simple random sampling using lottery method.

Purposive sampling technique was employed to identify participants for the qualitative in-depth interview. Interviewees were sought out until conceptual saturation was reached (i.e. until no new concepts were identified in successive interviews). Accordingly, the in-depth interview was held with a total of 10 interviewees from different colleges/institutes/school (agriculture and veterinary sciences, business and economics, medicine and health sciences, natural and computational sciences, law, social sciences and humanities, and technology). Besides differences in year of study, field of study and sex of interviewees, a good mix of participants with rich information were included in the study from students drawn from class representatives, participants of various HIV/AIDS and SRH intervention programs and trainings, trainees of gender-specific intervention programs and members of different clubs working on the problem under investigation like Anti-AIDS Club, Art Club and youth association.

### Variables and measurements

Variables pertaining to sexual history, risk perception, knowledge of STIs and contraceptive methods, and history of STIs and SRH problems were investigated and defined as follows:

- *Sexual history* was operationalized in terms of sexual intercourse ever and within the last 12 months, and age, educational level during and reason for the first sexual intercourse.
- *Risk perception* referred to whether respondents believed being at risk of getting HIV or not and their reasons for believing so.
- *Knowledge of STIs* represented whether or not respondents ever heard of STIs including HIV/AIDS, and were aware of their routes of transmission.
- *Knowledge of contraceptive methods* indicated awareness of contraceptive methods and their types, and source of information about the methods. Questions such as “do you know about modern contraceptive methods?” and “what are the contraceptive methods you are aware of?” were asked.
- *History of STIs and SRH problems* comprised respondents’ report of occurrence of any STIs within the last 12 months and of unwanted pregnancy and abortion ever. Respondents were asked questions such as “have you had any STIs in the past one-year?” and “have you ever obtained unwanted pregnancy?”

### Data collection

Survey was conducted through self-administered structured questionnaire. In-depth interviews were conducted using semi-structured interview guide by a sociologist with an experience of qualitative methodology and co-investigator. Different literatures were contacted and contextualized to develop the data collection tools. In order to control data quality, the questionnaire was pre-tested on 5% of the sample size among university students of another campus.

### Data analysis

The quantitative data was edited, coded and entered to SPSS version 20. Then double data entry verification was applied. Descriptive analysis (mean ± SD and median for continuous variables and frequencies and percentages for categorical variables) was conducted. The analysis steps of the data generated through in-depth interview involved categorically organizing and preparing the data, an initial reading and repeatedly reviewing through the information, continually coding the data, and developing from the codes a description and thematic analysis. Data from the quantitative and qualitative methods were first analyzed separately and then synthesized and analyzed in aggregate.

### Ethical considerations

Before data collection, ethical clearance was secured from the ethical review board of College of Medicine and Health Sciences of AU. The researchers were also aware of their obligation to respect the rights, needs, values, and desires of informants. To this end, the research objectives and data collection devices and activities were articulated, and informed consent was obtained.

## Results

### Socio-demographic profile

A total of 400 respondents participated in the survey, amongst whom 272 (68%) were male. The mean age of survey respondents was 21.14 ± SD 1.931. The majority (352, 88%) were single, and 269 (67.2%) were Oromo. The median monthly income of survey participants was 300 birr (Table 1). In the qualitative in-depth interview, there were a total of 10 interviewees (4 female and 6 male students). The interviewees were composed of different departments (natural resource management, public administration, nursing, chemistry, law, civics and ethical studies, English language and literature, electrical engineering, hydraulics engineering, and information technology).

**Table 1:** Socio-demographic profile of survey respondents in AU, January-February 2015 (*n* = 400)

Characteristics	*n* (%)
Sex	
Male	272 (68.0)
Female	128 (32.0)
Age (year)^a^	21.14 (1.931)
Marital status	
Single	352 (88.0)
In a relationship	33 (8.2)
Married	15 (3.8)
Ethnic group	
Oromo	269 (67.2)
Amhara	73 (18.2)
Tigre	21 (5.2)
Sidama	12 (3.0)
Guraghe	9 (2.2)
Other	16 (4.0)
Monthly income (Birr)^b^	300
Year of study	
First year	195 (48.8)
Second year	73 (18.2)
Third year	125 (31.2)
Fourth year	7 (1.8)

^a^mean (SD); ^b^median

### Sexual history

More than half, 212 (53%) of the survey participants ever had sexual intercourse, out of which 184 (86.8%) responded to subsequent questions regarding their age at, educational level during and reason for first sex. Accordingly, the mean age of their first sex was 17.29 ± SD 2.21 and nearly half, 86 (46.7%) were high school students (grade 9 to 12) when they had their first sexual intercourse. Followed by forced sex, 14 (7.6%), love was the main reason behind majority of survey respondents’, 137 (74.5%) first sexual practice. The survey data also indicated that 149 (37.2%) respondents had sexual intercourse within the last 12 months and their mean number of regular sexual partners within the specified period was 1.36 ± SD 0.505 (Table 2).

**Table 2:** Sexual history of survey respondents in AU, January-February 2015 (*n* = 400)

Characteristics	*n* (%)
Ever had sexual intercourse	
Yes	212 (53.0)
No	188 (47.0)
Age at first sex (year)^a^	17.29 (2.21)
Educational level during the first sexual intercourse^b^	
Elementary (Grade 1 – 8)	50 (27.2)
High school (Grade 9 – 12)	86 (46.7)
After joining university	48 (26.1)
Reason for the first sexual practice^b^	
Marriage	11 (6.0)
Love	137 (74.5)
To get some advantage from partner	12 (6.5)
Forced sex/rape	14 (7.6)
Other	10 (5.4)
Had sexual intercourse within the last 12 months	
Yes	149 (37.2)
No	251 (62.8)
Number of regular sexual partners within the last 12 months^a^	1.36 (0.505)

^a^mean (SD); ^b^n = 184

### Risk perception

Only 82 (21.1%) survey respondents perceived that they were at risk of HIV. For those who did not, their reasons for not being at risk were abstinence (162, 55.3%), faithfulness (90, 30.7%) and protected sex (32, 11%). Among participants of the survey who believed being at risk of HIV infection, 77 provided their reasons and 31 (40.3%) and 18 (23.4%) of them believed so because they had more than one sexual partner and had sex without condom, respectively (Table 3).

**Table 3:** Risk perception of survey respondents in AU, January-February 2015 (*n* = 400)

Characteristics	*n* (%)
Believe being at risk of getting HIV^a^	
Yes	82 (21.1)
No	306 (78.9)
Reason for being at risk of getting HIV^b^	
Had more than one sexual partner	31 (40.3)
Mistrust	10 (13.0)
Had sex without condom	18 (23.4)
Had sex with CSWs	1 (1.3)
Past history	6 (7.8)
Injury with contaminated sharp material	10 (13.0)
Blood transfusion	1 (1.3)
Reason for not being at risk of getting HIV^c^	
Abstinence	162 (55.3)
Faithfulness	90 (30.7)
Protected sex	32 (11)
Did not share injection	9 (3.1)

^a^ *n* = 388; ^b^ *n* = 77; ^c^ *n* = 293

In addition, lack of families’ control on their students in universities and students’ age of sexual intercourse were mentioned by in-depth interviewees as factors making exposure high. Moreover, a fourth year student raised peer pressure and the hidden instantaneous nature of sexual intercourse in the campus that led to sex without condom (in line with the quantitative data) as factors behind exposure to HIV/AIDS among students. He said:

“Students act in a way that exposes them to HIV, which can be due to peer pressure… Another is, for example, there can be lovers having sex… hiding from, among others, the campus police. They find themselves in hurry and may not have condom … which can expose them to the virus.”

### Knowledge of STIs and contraceptive methods

Majority (378, 94.5%) and almost all (392, 98%) of the survey respondents ever heard of STIs and HIV/AIDS, respectively. Most survey participants (355, 89.4%) knew modern contraceptive methods such as pills (241, 64.8%) and condoms (212, 56.8%), and their sources of information were, among others, the public health sector and mass/electronic media for 261 (69.8%) and 227 (60.9%) respondents, respectively (Table 4).

**Table 4:** Knowledge of STIs and contraceptive methods of survey respondents in AU, January-February 2015 (*n* = 400)

Characteristics	*n* (%)
Ever heard of STIs	
Yes	378 (94.5)
No	22 (5.5)
Ever heard of HIV/AIDS^a^	
Yes	392 (98.0)
No	6 (1.5)
Know modern contraceptive methods^b^	
Yes	355 (89.4)
No	42 (10.6)
Knowledge of types of contraceptives^c^	
Pill	241 (64.8)
IUCD	154 (41.4)
Injectable	156 (41.9)
Condom	212 (56.8)
Norplant	105 (28.2)
Diaphragm	82 (22.0)
Spermicides	63 (16.9)
Female sterilization	86 (23.1)
Male sterilization	83 (22.3)
Rhythm method	127 (34.8)
Source of information about contraceptive methods^c^	
Public health sector	261 (69.8)
Private health sector	121 (32.4)
Ambo university	93 (25.0)
Mass/electronic media	227 (60.9)
Print media	112 (29.9)
School	166 (44.6)
Spouse	26 (7.0)
Friend	76 (20.3)
Relatives	42 (11.3)

^a^ *n* = 398; ^b^ *n* = 397; ^c^multiple response question

Similarly, the in-depth interview result indicated that almost all participants knew STIs. The qualitative data analysis showed that HIV/AIDS, gonorrhea, syphilis and chancroid, in order of their count, were the major STIs repeatedly mentioned by interviewees. Regarding knowledge on STIs transmission, an interviewee explained that STIs including HIV/AIDS are “transmitted when there is blood contact, unsafe sexual intercourse, and from mother to child.” Besides, a second year male student asserted “… anything that can have contact with blood can transmit HIV/AIDS.” In-depth interview participants also rejected the notion of transmission by mosquito bites. In-depth interviewees’ source of information about STIs was mainly from in-campus trainings, and also from peers, media, previous classes in high school and religious teachings given occasionally. For instance, a second year male student said:

“I used to know STIs roughly based on boring information I used to hear from TVs and radios and informal talks with friends. But later I started to know more when it was in the form training, which was wider and deeper.”

### History of STIs and SRH problems

More than one fifth of survey respondents (91, 22.8%) had any of the STIs in the past one year. In terms of SRH problems among girls, 6 (5%) out of 120 ever obtained unwanted pregnancy and 3 (2.5%) out of 119 ever aborted unwanted pregnancy (Table 5). The in-depth interviewees similarly agreed that STIs were challenges of AU students. One interviewee even shared the experience of his female friends in relation to STIs, particularly gonorrhea, stating “…there were three female friends of mine who told me that they had gonorrhea. I think a lot of girls are affected by this disease.”

**Table 5:** History of STIs and SRH problems among survey respondents in AU, January-February 2015 (*n* = 400)

Characteristics	*n* (%)
Had any of STIs in the past one year	
Yes	91 (22.8)
No	309 (77.2)
Ever obtained unwanted pregnancy (Girls)^a^	
Yes	6 (5.0)
No	114 (95.0)
Ever aborted unwanted pregnancy (Girls)^b^	
Yes	3 (2.5)
No	116 (97.5)

^a^ *n* = 120; ^b^ *n* = 119

Female in-depth interview participants reported that unwanted pregnancy was one of the major SRH problems among female students of the main campus. A fourth year law student, telling that unwanted pregnancy happened due to lack of caution, said “it is really a problem… there are many cases of unwanted pregnancy, we hear about it around our dormitories.” Upon probing about its prevalence, she confessed, “to tell you the truth, I know at least seven students with unwanted pregnancy out of twenty female students.” Another female interviewee also told that unwanted pregnancy was commonly encountered and reasoned “the fact that there is unsafe sex implies higher probability of the occurrence of unwanted pregnancy.” Like her seniors, a hydraulics engineering first year student similarly reported that she saw three pregnant students in her stay at the campus for only a semester.

With regard to abortion, most female interviewees believed that unsafe abortion was an issue of great concern among female students of the main campus. Accordingly, telling that unsafe abortion among campus female students was a serious problem, a female student exposed that:

“We hear most of the time when students have unsafe abortion or encounter complications as a result. Although it is not explicitly observed, there are rumors that many cases of unsafe abortion are undertaken in secret… There are even cases I am aware of. We were taking a certain course and some people who render abortion services, who were also participants of the course, told us there are many cases of abortion.”

## Discussion

Findings of the study indicated that more than half of survey participants (53%) ever had sexual intercourse, which was higher than previous percentages reported in the same university in 2011, 2013 and 2015 (39%, 42.8% and 40.8% respectively) [6, 8, 9]. With the mean age during first sex of 17.29 ± SD 2.21, nearly half of the students were in high school when they had their first sexual intercourse mainly due to love. Respondents also mentioned forced sex as a reason for first sex which may be a reason for continued sexual experiences.

Only 21.1% of survey respondents in this study perceived that they were at risk of HIV. In comparison, 80.7% of students in Addis Ababa University and over half (56.3%) of students who practiced unsafe sex in Jimma University did not perceive themselves as being exposed to risk [1, 7]. Almost all survey respondents ever heard of STIs and HIV/AIDS, and most knew modern contraceptive methods. In line with the quantitative result, the qualitative data showed that almost all participants knew STIs, and HIV/AIDS, gonorrhea, syphilis and chancroid were the major STIs mentioned by interviewees.

In line with the qualitative finding that STIs were problems among the students, the survey data indicated more than one fifth (22.8%) had any of the STIs in the past one year. Correspondingly, 6.3% of junior students reported history of STIs in another study in the same university [9]. Although the quantitative data in the present study showed unsafe abortion existed in the campus minimally, the qualitative data revealed significant cases of unwanted pregnancy and unsafe abortion were observed and affirmed they were major reproductive health problems among female students. Reasons for unwanted pregnancy and unsafe abortion may extend beyond non-use of contraceptive methods, as respondents mentioned forced sex/sexual violence as a reason for first sex.

The results of this study should be viewed in light of its limitations. There were some mismatches between the qualitative and quantitative results in this study, particularly regarding SRH problems. The inconsistency between the two types of data could be explained by the fact that the in-depth interviewees were asked about what they saw in the campus in general, not about their own specific cases. So, they freely reported about it. Survey respondents, on the other hand, were asked about their own cases. Given the secretive nature of the practice and the informal negative sanctions such as ridicule and gossip it entailed, it was more likely that they preferred to hide it. Due to financial constraints the study area was also limited to the main campus only, making generalizations about the whole university difficult.

## Conclusions

SRH/STIs were problems among students of the university. Although students knew about STIs, they failed to apply their knowledge to themselves and their sexual health. Generally, the quantitative results demonstrated high levels of knowledge and accurate risk perceptions, but the qualitative suggested that poor health outcomes such as unwanted pregnancy and unsafe abortion were more common than was reported in the quantitative survey.

Although the concepts investigated are not new, the study provides a significant contribution to the current body of knowledge as it is set in a lower income country where SRH issues among youth is still a major public health problem. The findings have relevance to healthcare providers and public health professionals who may be seeking to implement interventions within low-resource settings to address and improve SRH outcomes.

Intervention programs to contain STIs and SRH problems should start from high school because majority of the students started sexual intercourses at high school with an early age sexual experience. Efforts to reduce sexual violence must also be strengthened because forced sex is still the reason behind first sexual intercourse for many students. Since the qualitative result indicated that the information transmitted about STIs and SRH via the media was found to be boring, such programs should be youth friendly and interesting. There is also a need for increasing STI service availability on campus. The university needs to launch a separate program directed towards STIs and SRH problems especially among female students. Further researches also need to be conducted on SRH problems among students of other campuses.

## Abbreviations

AU: Ambo University
HEIs: Higher education institutions
SRH: Sexual and reproductive health
STIs: Sexually transmitted infections
